# A Rare Case of Tongkat Ali-Induced Liver Injury: A Case Report

**DOI:** 10.7759/cureus.56639

**Published:** 2024-03-21

**Authors:** Aboud Kaliounji, Grace Shadid, Helena Saba, Sushil Ahlawat

**Affiliations:** 1 Internal Medicine, SUNY (State University of New York) Downstate Health Sciences University, New York, USA; 2 Gastroenterology and Hepatology, SUNY (State University of New York) Downstate Health Sciences University, New York, USA

**Keywords:** nutraceuticals, anabolic supplements, testosterone, herbal-induced liver injury, drug-induced liver injury, tongkat ali

## Abstract

Drug-induced liver injury (DILI) presents a significant challenge in clinical practice, particularly with the rising popularity of herbal and dietary supplements (HDS) in the United States. Tongkat Ali (Eurycoma longifolia Jack), a Southeast Asian herb, has garnered attention for its purported health benefits, including enhancing testosterone levels. Here, we present a case of a 47-year-old male with acute liver injury following Tongkat Ali use, the first reported case of its kind in the literature. The patient exhibited worsening scleral icterus, elevated liver enzymes, and jaundice shortly after initiating Tongkat Ali supplementation, prompting hospitalization and subsequent clinical improvement upon discontinuation of the supplement. Differential diagnosis and exclusion of other etiologies were essential in establishing the causal link between Tongkat Ali consumption and liver damage, underscoring the difficulty in diagnosing HDS-induced liver injury. The rise in DILI cases parallels the expanding use of nutraceuticals, necessitating vigilance among healthcare professionals. While mechanisms of herbal-induced liver injury remain unclear, genetic predisposition and metabolic factors may be implicated. This case emphasizes the importance of heightened awareness among healthcare providers regarding the potential hepatotoxic effects of herbal supplements, particularly in individuals consuming multiple agents. Further research into the safety profile and mechanisms of Tongkat Ali-induced liver injury is warranted to inform clinical management and promote safer supplement use.

## Introduction

Drug-induced liver injury (DILI) is a major adverse effect of herbal supplement use that can lead to acute liver failure and, in severe cases, death. DILI is the most common cause of acute liver failure in the Western world [1]. This form of liver injury is challenging to diagnose, as its cause can be multifactorial, developing from the use of a wide range of medications, including prescription drugs and nutraceuticals [2]. The incidence of DILI is estimated to be 14-19 cases per 100,000 persons [3]. In recent years, herbal and dietary supplements (HDS), including bodybuilding agents, have become increasingly popular for their health benefits but have also led to an increased number of liver injuries, becoming the second most common cause of liver injury [4]. Tongkat Ali is one example of these herbal supplements; however, there are no reports of Tongkat Ali-induced liver injury in the literature.

Tongkat Ali, also known as Eurycoma longifolia Jack, is an herb native to Southeast Asia [5]. The plant has been used in traditional medicine for its antimalaria, anti-diabetic, anti-microbial, and antipyretic properties. In addition, regular intake of the plant’s root extract is believed to enhance testosterone levels in men, thus making the herb popular among bodybuilders. Here, the first case of liver injury secondary to Tongkat Ali use is presented.

## Case presentation

**Figure 1:**
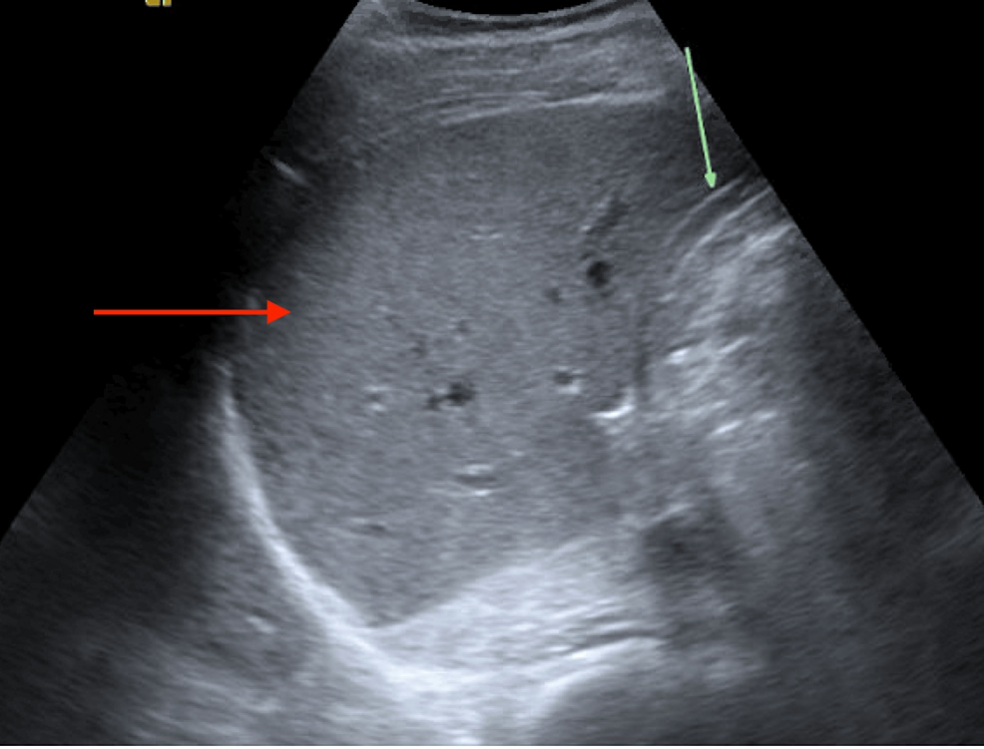
Right upper quadrant ultrasound showing a normal-sized-liver with normal echogenicity (red arrow); the green arrow shows a contracted gallbladder with wall thickening

A 47-year-old male with hypertension and a tobacco use history presented to the emergency room (ER) with worsening scleral icterus and dark urine for three days. He complained of nausea, vomiting, and generalized abdominal pain the week prior for three days that he contributed to “food poisoning”. On arrival, his blood pressure was 142/100 mmHg; heart rate (HR) of 70 bpm; SpO_2_ of 99%, and a temperature of 98.2 °F. Physical exam was significant only for bilateral scleral icterus. His laboratory tests were significant for elevated liver function tests: aspartate aminotransferase (AST): 234 U/L, alanine transaminase (ALT): 470 U/L, alkaline phosphatase (ALP): 192 U/L, and total bilirubin: 7.5 mg/dL. His international normalized ratio (INR) was within normal limits at 1.0. Hepatitis A antibody immunoglobulin M (IgM), hepatitis B surface antigen, hepatitis B core IgM, and hepatitis C Ab were all non-reactive. Hepatitis C virus (HCV) RNA was undetectable. Urinalysis demonstrated dark brown urine positive with a large amount of bilirubin. Right upper quadrant ultrasound showed no duct dilation or hepatic morphological abnormalities (Figure 1). The patient refused to be admitted and was referred to follow up with gastroenterology (GI) as an outpatient.

At his GI appointment, four days after his ER visit, he had additional complaints of anorexia, postprandial upper abdominal discomfort, and early satiety with approximately 15-pound weight loss. He denied recent travel and sick contacts and was up to date on all vaccinations. Upon further questioning, he endorsed starting a new herbal supplement for bodybuilding one week prior to the onset of symptoms: Tongkat Ali. He drank wine socially, was in a monogamous relationship with a woman, and did not take any other medications or drugs. The patient was advised to go to the ER for further workup.

**Figure 2:**
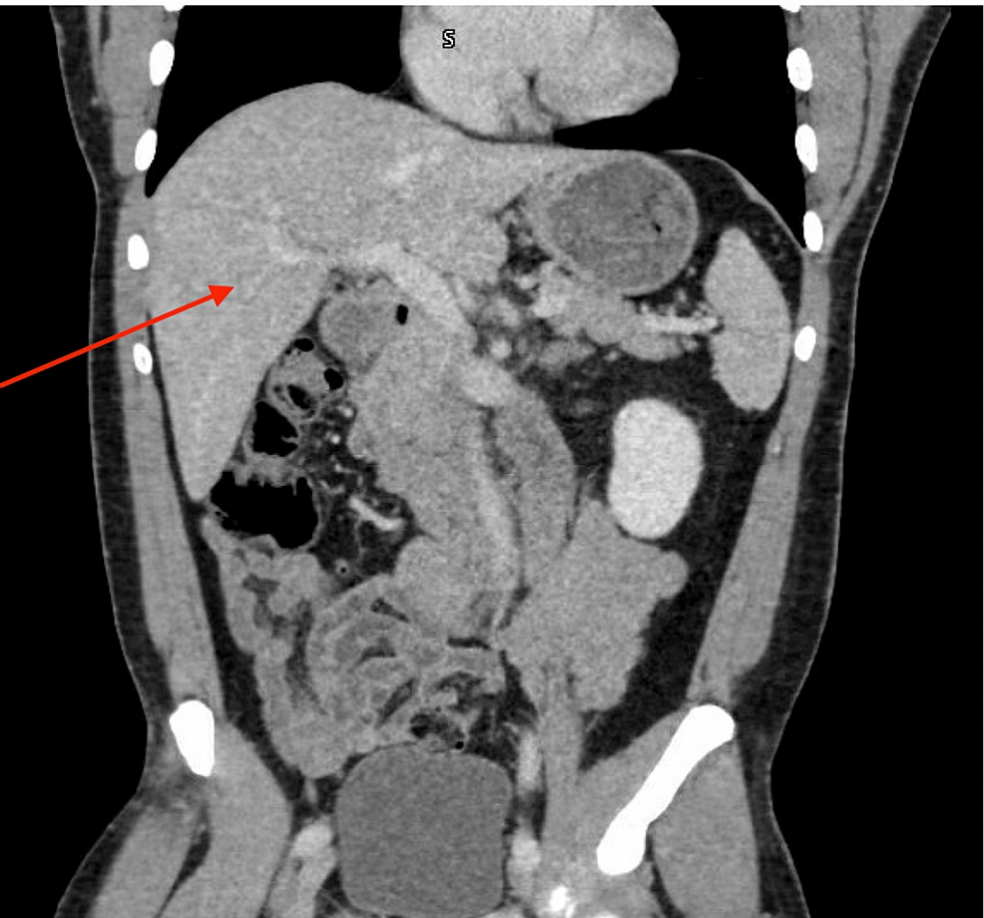
Computed tomography (CT) abdomen/pelvis with contrast showing no hepatobiliary pathology; red arrow shows a normal-sized liver without any masses, nodules, or fibrotic changes.

In the ER, he was hemodynamically stable. Physical exam was significant for scleral icterus and worsening jaundice. The blood test revealed AST: 445 U/L, ALT: 876 U/L, ALP: 238 U/L, total bilirubin: 14.3 mg/dL, and a direct bilirubin of 7.78 mg/dL (Table 1). Iron studies showed an iron level of 180 μg/dL, TIBC of 346 ug/dL, and an elevated ferritin of 2249 ng/mL. ANA, anti-smooth antibodies, and anti-mitochondrial antibodies (AMA) were negative. Ceruloplasmin was mildly elevated at 45 mg/dL. Screening for HIV, syphilis, chlamydia, and Neisseria gonorrhoeae was negative. Right upper quadrant ultrasound with Doppler demonstrated patency of the hepatic vasculature and no signs of liver cirrhosis. Murphy's sign was negative. CT abdomen/pelvis demonstrated a normal liver in both size and contour without any nodules or suspicious lesions (Figure 2). There was also no intra or extra-hepatic biliary duct dilation.

**Table 1:** Trend of liver enzymes and total bilirubin * First ER visit ** Follow-up visit post-discharge AST, ALT, and ALP were not measured on day 5. AST: aspartate aminotransferase; ALT: alanine aminotransferase; ALP: alkaline phosphatase

Day#	0*	4	5	6	7	24**
ALP (U/L)	192	238		220	235	157
AST (U/L)	234	445		303	230	74
ALT (U/L)	470	876		744	647	193
Total bilirubin (mg/dL)	7.5	14.3	13.1	13.2	12.9	5.3

Throughout his hospitalization, his liver enzymes down-trended, as shown in Table 1. His physical exam was significant for scleral icterus and improved jaundice. After an uncomplicated four-day hospitalization, he was advised to stop his new herbal supplement and was discharged with a GI and primary care physician (PCP) follow-up. The patient followed up with his PCP two weeks following discharge with markedly symptomatic improvement in his jaundice, scleral icterus, and urine color. His liver enzymes continued to down-trend.

## Discussion

Over the past few decades, the use of nutraceuticals in America has increased due to their popularity and lack of regulation. This increased consumption was found to be associated with a rise in DILI incidence, from 7% to 20% between 2004 and 2012 [4]. The FDA has approved many drugs that contain testosterone and androgenic steroids for sex hormonal therapy and other medical conditions, which have been illicitly used for performance enhancement and bodybuilding. While many herbs are generally considered safe when used appropriately, some have been associated with liver toxicity and herbal-induced liver injury (HILI). The liver plays a crucial role in metabolizing drugs and toxins, and some individuals may be more susceptible to liver injury due to genetic factors or pre-existing liver conditions. Herbal-induced liver injury accounts for 20% of hepatotoxicity cases and 13% of acute liver failure cases in America, with recent studies estimating its incidence rate to be 1.16 per 100,000 people [6]. In a prospective study from the drug-induced liver injury network looking at 130 cases of HDS-related liver injury, at least 45 were attributed to bodybuilding agents, many of which contain anabolic steroids with synthetic derivatives of testosterone [7].

The clinical course of HILI is heterogeneous and ranges from asymptomatic transaminitis and hepatocellular or cholestatic jaundice that can resolve completely after suspending the offending agent to fulminant liver failure requiring urgent liver transplant [8]. In the case presented here, the patient's R- Factor was greater than 5, on both day 0 and day 4 presentation to the ER, suggestive of a hepatocellular pattern of liver injury. Confirming the diagnosis can be challenging and requires a comprehensive medical history and a high index of suspicion. While establishing a correlation between the onset of liver disease and the time of drug exposure is critical in making the diagnosis, it is imperative to rule out other etiologies such as infection and auto-immune diseases that often warrant a liver biopsy. Since there still lacks a valid diagnostic marker to diagnose DILI, the medical community developed scales, such as The Rousse Uclaf Causality Assessment Method of the Council of International Organization of Medical Science (RUCAM/CIOMS), to help establish the cause of liver injury.

Further contributing to the challenge, patients often take multiple supplements, making it difficult to pinpoint a single offending agent. For instance, Peri et al. reported two cases of acute hepatitis secondary to herbal supplements [9]. The first patient was consuming Corydalis for 18 months for its analgesic effect while the second patient was taking a supplement with multiple compounds, including Tongkat Ali root (Eurycoma longifolia), as well as saw palmetto fruit extract and horny goat weed, of which the latter two have been implicated with liver injury. In such instances, it is extremely difficult to determine the agent that is responsible for hepatic injury.

Tongkat Ali, an herbal medicinal plant that is also known as Long Jack, Malaysian Ginseng, or Local Ginseng, is often used as a traditional remedy for various health purposes, including as an aphrodisiac and to boost energy and testosterone levels. It is rich in various bioactive compounds, such as pasakbumin-B, eurycomanone, eurycomalactone, eurycolactone, eurycomaoside, laurycolactone, squalene derivatives, and canthin-6-one alkoids, among which alkaloids and quassinoids form a major portion [5]. The case presented here is believed to be the first reported Tongkat Ali-induced liver injury in the literature to date.

While the active compounds in Tongkat Ali, such as quassinoids, are metabolized in the liver through various enzymatic processes, the pathogenesis of Tongkat Ali-induced liver injury is not completely understood. Drug-induced liver injuries are thought to occur via several mechanisms, including direct toxic effect or production of metabolites leading to impairment or alteration of the structural, functional, or integrity of the liver, systemic hypersensitivity reaction, and immune-mediated liver damage [10]. Moreover, researchers have found considerable evidence that people with specific genetic factors are more susceptible to DILI than others [11]. Urban et al. reported that the human leukocyte antigen (HLA) genotype is a risk factor for DILI with a wide range of drugs as well as some drug metabolism genes such as NAT2 and UGT2B7 [11]. There is ongoing research to better understand the mechanism of mitochondrial injury, the role of the immune system, and the impairment of bile salt excretion [12].

Cases of steroid-induced liver injury in the literature follow a similar clinical presentation, including pronounced jaundice and pruritus in middle-aged men, particularly bodybuilders one to six months after initiating the supplement [7]. Interestingly, the patient experienced jaundice after only one week of Tongkat Ali intake, with no associated pruritus, which can be explained by the early phase of bilirubinemia compared to other reported cases in the literature with a reported bilirubin of 40-50 mg/dL. The overall clinical picture of steroids-induced liver injury cases, including lab tests, clinical presentation, and liver histology previously demonstrated canalicular cholestasis with minimal inflammation and necrosis of the hepatocytes. The exact mechanism of this pattern of injury is not well understood but suggests impairment of canalicular function rather than hepatocellular or cholangiocytic liver damage.

## Conclusions

While there are limited studies evaluating the safety and toxicity of Tongkat Ali in animals, there remains a deficiency in research examining the long-term safety and effectiveness of Tongkat Ali. Physicians and other professionals must be vigilant and informed about the potential adverse effects associated with its use. This case raises awareness among the general public and healthcare providers regarding the potential risks of herbal supplements, especially in young men who tend to consume anabolic supplements.
